# A resource of “bottom-line” variant associations for 1,281 complex traits by integrating data across published genome-wide association studies

**DOI:** 10.21203/rs.3.rs-8585052/v1

**Published:** 2026-01-22

**Authors:** Trang Nguyen, Furkan Büyükgöl, Patrick Smadbeck, Jeffrey Massung, Maria C. Costanzo, Monica Ruiz, Peter Dornbos, Satoshi Yoshiji, Ryan Koesterer, Thanh Long Nguyen, Dongkeun Jang, Quy Hoang, Yue Ji, Aoife McMahon, Sebanti Sengupta, Xianyong Yin, Brady Ryan, Ryan P. Welch, Jorien Treur, Connie R. Bezzina, Goncalo Abecasis, Michael Boehnke, Noël P. Burtt, Jason Flannick

**Affiliations:** 1 Programs in Metabolism and Medical & Population Genetics, The Broad Institute of MIT and Harvard, Cambridge, MA, USA; 2 Department of Clinical and Experimental Cardiology, Heart Center, Amsterdam Cardiovascular Sciences, Cardiomyopathy and Arrhythmia, Amsterdam UMC, location University of Amsterdam, Amsterdam, The Netherlands; 3 Genetic Epidemiology, Department of Psychiatry, Amsterdam UMC, University of Amsterdam, The Netherlands; 4 Department of Pediatrics, Dr. von Hauner Children's Hospital, LMU University Hospital Munich, Munich, Germany; 5 Institute of Translational Genomics, Computational Health Center, Helmholtz Zentrum München – German Research Center for Environmental Health, Neuherberg, Germany; 6 Division of Genetics and Genomics, Boston Children’s Hospital, Boston, MA, USA; 7 Department of Pediatrics, Harvard Medical School, Boston, MA, USA; 8 Regeneron Genetics Center, Tarrytown, New York, USA; 9 Department of Human Genetics, McGill University, Montréal, Québec, Canada; 10 European Molecular Biology Laboratory, European Bioinformatics Institute, Wellcome Genome Campus, Hinxton, Cambridge CB10 1SA, UK; 11 Department of Biostatistics and The Center for Statistical Genetics, University of Michigan, Ann Arbor, MI, USA; 12 Department of Epidemiology, School of Public Health, Lianyungang Medical-Education Innovation Center, Nanjing Medical University, Nanjing, China; 13 Jiangsu Key Laboratory of Molecular Targets and Intervention for Metabolic Diseases, Nanjing, China

## Abstract

Through an analysis of 2,602 genome-wide association studies (GWAS) across 830 human traits, we find that most (56% of) well-studied traits have at least two published GWAS, and many (29%) have at least five. We show that the lack of an established approach for adjudicating variant association estimates across multiple published studies can lead to uncertainty and invalid inferences: using all associations ever published for a trait increases true positives (by 12%) but also false positives (by 55%) relative to using associations from the largest published GWAS for the trait. We employ a “bottom-line” procedure for meta-analyzing published GWAS while inferring and accounting for sample overlap, which identifies a more accurate and comprehensive list of associations relative to existing approaches. Five commonly used bioinformatic methods for post-GWAS analyses produce reliable results when applied to the bottom-line associations. We present these results for 1,281 human complex traits, including 1,839 single-ancestry and 576 trans-ancestry analyses, for browsing or download via the NHGRI Association to Function Knowledge Portal. This resource of “consensus” GWAS results is intended to increase replicability, reuse, and interpretation of GWAS and downstream analyses.

## Introduction

Genome-wide association studies (GWAS) provide the most comprehensive resource of complex trait genetic associations, which have uses ranging from disease gene discovery^1,2^ to genetic risk prediction^1,3^ to drug target validation^1,4^. Over time, many traits have had many GWAS published – for example, 169 GWAS for type 2 diabetes and 301 for low-density lipoprotein cholesterol levels^5^. When multiple GWAS have been conducted for the same trait, it is often unclear what the “consensus” estimate of association is for each genetic variant, which poses practical challenges when enumerating the signals that exceed genome-wide significance or applying “post-GWAS” statistical methods^6^.

In practice, researchers have employed several (often implicit) approaches to obtain consensus association estimates when multiple GWAS for the same trait are available. One approach is to use the most “comprehensive” published GWAS, which requires balancing criteria for comprehensiveness such as sample size, number of analyzed variants, specificity of disease definition, and ancestry inclusion – a choice further complicated when whole-exome and whole-genome sequencing studies (which include far more variants than array-based GWAS but usually with far smaller sample sizes) are available. Using results from only one available GWAS might also lead to false negative associations, either because a variant achieves significance in a GWAS other than the selected one or because the selected GWAS has excluded a substantial number of variants in order to maximize sample size^7^. A second approach for obtaining consensus association estimates (common when evaluating a gene’s “genetic support”^8^ or an experimental finding^9^) is to search the literature or association databases^5,10^ to see if a significant association has ever been reported for the variant. This might unknowingly lead to “cherry-picking” and a consequent excess of false positive associations. A final, more statistically valid approach is to meta-analyze available studies. In the typical case when GWAS are not independent^11,12^, most meta-analysis methods require study-level statistics to first be re-estimated after removing overlapping samples, which is feasible but potentially challenging for large-scale GWAS consortia^6,13^ with access to individual-level data and difficult for an investigator with access only to published summary statistics. While it may be possible to defend one or more of these consensus estimation approaches in a particular situation, different approaches taken across studies collectively reduce power, comparability, and reproducibility.

**Figure 1.**
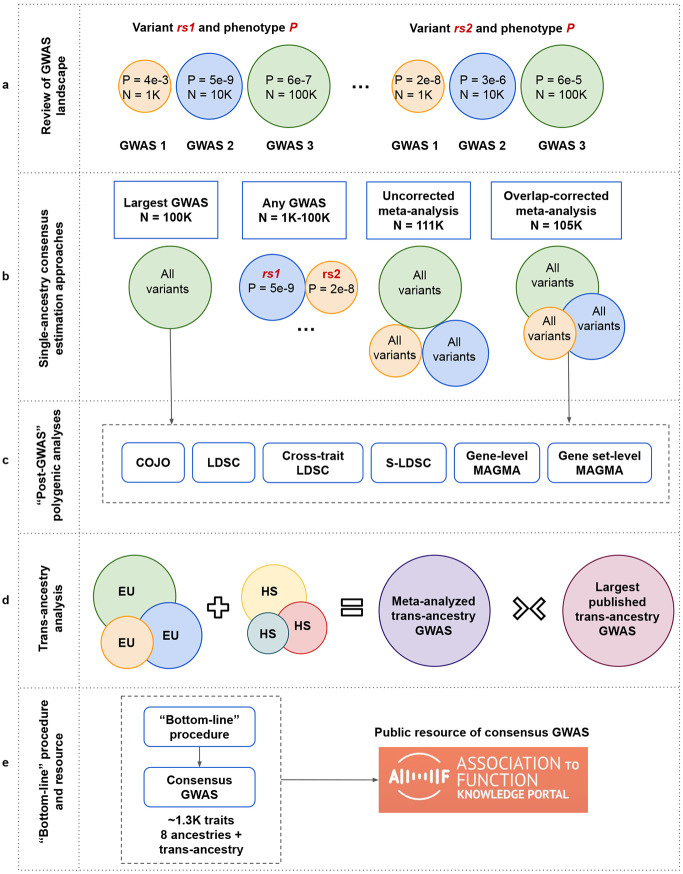
Overview of the study. **(a)** Review of the number of GWAS per trait. **(b)** Different approaches for estimating the single-ancestry consensus association signals. **(c)** Comparison of “post-GWAS” analyses using the largest GWAS vs. the overlap-corrected GWAS. **(d)** Evaluation of trans-ancestry association signals against the largest published trans-ancestry GWAS. EU (European) and HS (Hispanic or Latin American) are two example ancestries. **(e)** The “bottom-line” procedure and the resource of consensus GWAS publicly available on the A2FKP.

Here, we compare and quantify errors that result from existing approaches for obtaining consensus variant association estimates across published GWAS, through an analysis of 2,602 studies of 830 traits across 9 ancestries (Fig. 1). We design and apply a new procedure to produce “bottom-line” estimates of association for each variant across all traits, which employs meta-analysis of published studies while statistically inferring and accounting for sample overlap (Sengupta et al. in preparation)^14^. We show that the bottom-line procedure produces more comprehensive and accurate lists of associations signals than do existing approaches and demonstrate the suitability of the bottom-line summary statistics for downstream post-GWAS analytical methods. The bottom-line associations for 1,281 traits are publicly available on the NHGRI Association to Function Knowledge Portal (A2FKP; https://a2fkp.org) for browsing and download. We propose the bottom-line results as a resource of consensus GWAS associations to increase replicability, reuse, and meaningful interpretation of GWAS results.

## Results

### Many traits have multiple published GWAS

**Figure 2.**
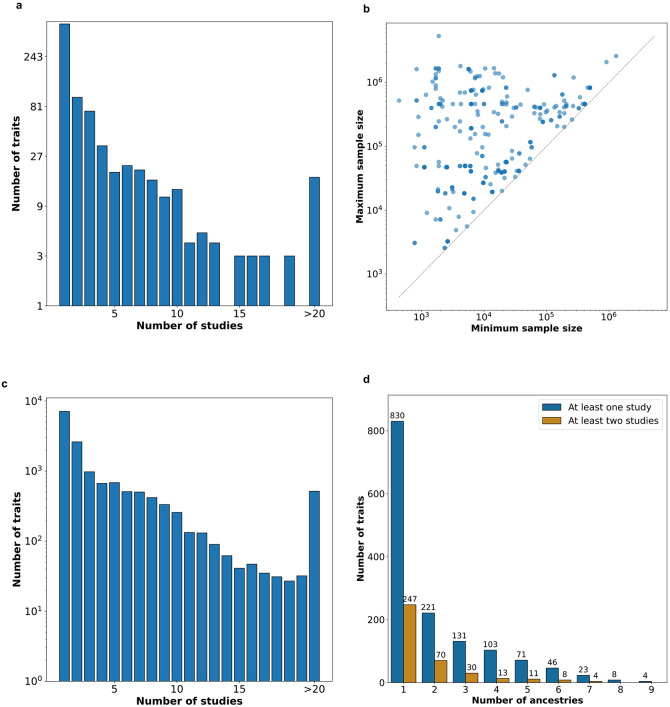
Summary of GWAS available for various traits. **(a)** Number of traits with different numbers of studies available across 830 traits in the A2FKP. **(b)** Minimum sample size vs. maximum sample size of studies across 261 trait-ancestry pairs with more than one study in the A2FKP. **(c)** Number of traits with different numbers of studies available across 15,217 traits in the GWAS Catalog. **(d)** Number of traits with at least one study available in (at least) one to nine ancestries (blue) and number of traits with at least two studies available in (at least) one to nine ancestries (orange) across 830 traits in the A2FKP.

To determine how common it is for traits to have multiple published GWAS, we analyzed 2,602 summary statistic datasets for 830 traits within the A2FKP, a genetics knowledge portal that encompasses 16 community-created genetics knowledge portals^15-19^ and which aims to capture all community-approved GWAS summary statistics across a wide range of traits (**Supplementary Table 1;** Methods). On average, traits in the A2FKP had 3.1 studies available, with 332 (40%) traits having at least two studies available and 129 (15.5%) having at least five (Fig. 2a; **Supplementary Table 1**). Restricting the analysis to 331 well-studied traits (defined as having at least one study of >100K samples), these numbers increased to 5.2 studies per trait, with 55.9% of traits having at least two published GWAS and 29% of traits having at least five. Sample sizes varied widely across studies for most traits, with an average 84-fold difference between the largest and smallest studies (Fig. 2b; **Supplementary Table 1**). As the A2FKP is a selective resource (Methods), these values understate the actual number of GWAS published for a typical trait: across the 15,217 traits in the EBI/NHGRI GWAS catalog, the average number of studies is 6.8 (53% of traits have at least two, and 25% have at least five; Fig. 2c; **Supplementary Table 2**). In other words, genetic variants have on average nearly seven different published association estimates per trait.

Within the A2FKP, traits had multiple published studies for two reasons. First, 221 (27% of) traits had studies conducted within different ancestries (Fig. 2d; **Supplementary Table 1**). Statistically combining studies in different ancestries through meta-analysis is straightforward, as such studies include (presumably) independent samples, but interpreting the resulting associations is challenging due to potential allelic heterogeneity across ancestries. Second, 247 (30% of) traits had at least two studies conducted within at least one ancestry (Fig. 2d; **Supplementary Table 1**). In these situations, statistically combining studies is non-trivial, as samples may overlap, but interpreting associations is more straightforward compared to trans-ancestry analyses. In total, 136 (16% of) traits fell into both categories. We conclude that with GWAS now widespread, the need for an approach to reconcile multiple association estimates into one consensus estimate for a variant has become acute.

### Two common approaches to estimate single-ancestry consensus associations across published GWAS are prone to statistical errors

Perhaps the most common use of GWAS results is to produce a list of association signals for a trait. We compared two approaches to produce such a list from multiple GWAS: (a) using the associations significant (*P* < 5×10^−8^) in the largest GWAS and (b) using the associations significant in any published GWAS (Methods). For each approach, we conservatively defined a significant “association signal” as a lead single-nucleotide polymorphism (SNP) together with all variants in detectable linkage disequilibrium (LD; r^2^ > 0.01) with it (using PLINK’s clumping algorithm), and we conservatively considered two signals to be the same if their variant sets overlapped (Methods). We restricted our analysis to common variants (MAF > 5%), since they account for the majority of GWAS locus discoveries.

**Figure 3.**
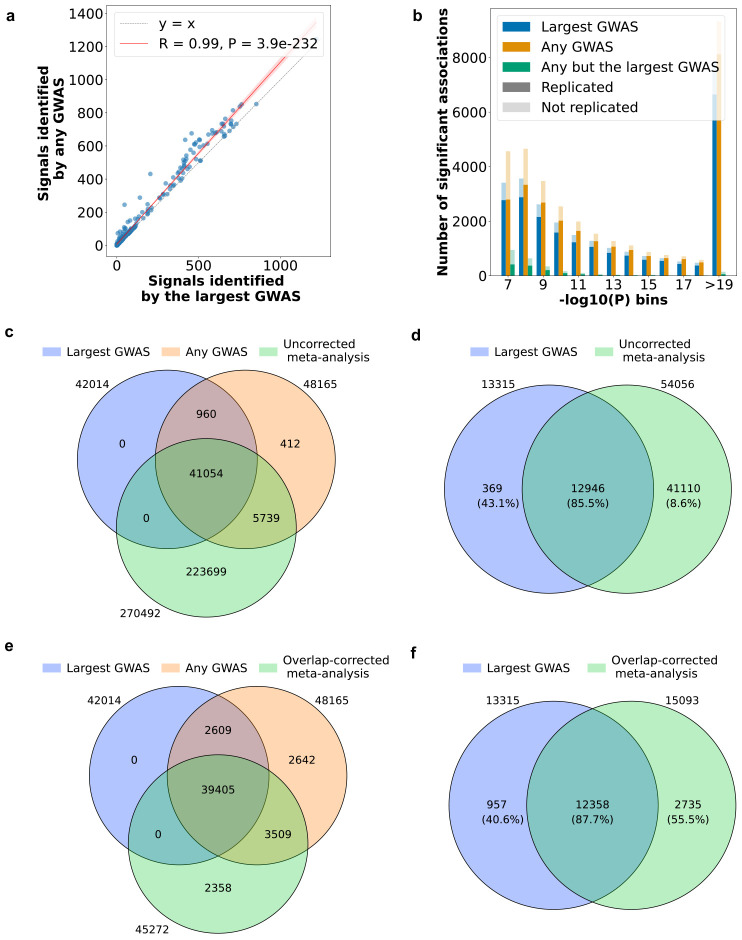
Comparison of single-ancestry association signals produced by four consensus estimation approaches. **(a)** Number of association signals significant in the largest GWAS vs. any GWAS. **(b)** Number of association signals (original clumps instead of the merged clumps) significant in the largest GWAS (blue), any GWAS (orange), and any GWAS but not the largest GWAS (green) at various significance levels. Dark areas: signals replicated in the gold-standard GWAS, light areas: signals not replicated in the gold-standard GWAS. Numbers on top of bars represent the proportion of replicated signals. **(c)** Overlap of association signals significant in the largest GWAS (blue, top-left), any GWAS (orange, top-right) and uncorrected meta-analysis (green, bottom). **(d)** Overlap of association signals significant in the largest GWAS (blue, left) and uncorrected meta-analysis (green, right). Percentages represent the proportions of signals replicated in the gold-standard GWAS. **(e)** Overlap of association signals significant in the largest GWAS (blue, top-left), any GWAS (orange, top-right) and overlap-corrected meta-analysis (green, bottom). **(f)** Overlap of association signals significant in the largest GWAS (blue, left) and overlap-corrected meta-analysis (green, right). Percentages represent the proportions of signals replicated in the gold-standard GWAS. (a), (c), (e): Main analysis of 270 trait-ancestry pairs, (b), (d), (f): Validation analysis of 97 trait-ancestry pairs.

Focusing first on single-ancestry analyses, for the 270 trait-ancestry pairs (across 207 traits and 7 ancestries) with at least two studies available in the A2FKP (**Supplementary Table 3a**), the “any GWAS” approach produced 14.6% more association signals than did the “largest GWAS” approach (22.8 more associations per trait-ancestry pair; Fig. 3a; **Supplementary Table 3a**). As expected, trait-ancestry pairs with more studies tended to have a larger increase in associations, although this trend was not statistically significant (*P* = 0.08; **Supplementary Fig. 1a**). A substantial fraction of these additional associations (22.3%) was for variants absent from the largest GWAS, presumably because they were not genotyped or imputed – this underscores that the largest available GWAS is not necessarily the most comprehensive in terms of the number of variants studied. The remaining additional associations achieved significance in smaller but not necessarily small studies: the GWAS that detected these associations was typically (80% of the time) at least half the size of the largest GWAS and was only rarely (2.4% of the time) an order of magnitude smaller than the largest GWAS (**Supplementary Fig. 1b**). Moreover, for the vast majority (93%) of the additional associations, there were multiple studies in which the unique association achieved *P* < 0.05 (**Supplementary Fig. 1b**). One example is an association between a variant nearby *COL6A3* and coronary artery disease (CAD), which was not reported as genome-wide significant in the largest GWAS^20^ despite being reported in previous studies^21-23^ that highlighted COL6A3-derived endotrophin as a potential therapeutic target for CAD^24^.

To estimate how many of these additional associations found by the “any GWAS” approach are reproducible associations (as opposed to study-specific noise), we conducted a retrospective leave-one-out validation analysis, in which we removed the largest GWAS from each trait-ancestry pair as a source of “gold-standard” association signals and generated two lists of associations from the remaining studies according to the “largest GWAS” or “any GWAS” approach (Methods). This analysis included 80 traits and 97 trait-ancestry pairs with at least two remaining studies (**Supplementary Table 3b**) and 23,109 gold standard association signals.

In this validation analysis, the “any GWAS” approach produced 15,815 association signals, 2,500 (18.8%) more than the largest GWAS **(Supplementary Table 3b)**. However, only 54.4% of these additional signals were “replicated” (i.e., also genome-wide significant) in the gold-standard set, compared to 84.3% of signals identified by the largest GWAS (**Supplementary Fig. 1c**). The lower replication rate of the additional signals was almost entirely due to associations of borderline significance (e.g. with *P*-values just below 5×10^−8^; Fig. 3b). As a result, even though the “any GWAS” approach had a 12% increase in true positive significant associations relative to the largest GWAS, it also had a 55% increase in false positive associations relative to the largest GWAS. These numbers quantify the expected result that considering associations significant in any GWAS produces more comprehensive but less accurate association lists relative to considering only those significant in the largest published GWAS.

### Overlap-corrected single-ancestry meta-analysis of published GWAS mitigates type I and type II errors

The recommended approach to combine published findings is via meta-analysis^25,26^, and we explored whether a similar strategy could work for combining published GWAS associations. For each of the 270 trait-ancestry pairs with more than one GWAS available, we conducted a fixed-effects meta-analysis across all studies for common variants (termed “uncorrected meta-analysis” due to its ignorance of sample overlap; Methods). The uncorrected meta-analysis produced 270,492 associations, most (82.7%) of which were not found in any single published GWAS (Fig. 3c; **Supplementary Table 3a**). In the leave-one-out validation analysis, the association signals significant in the uncorrected meta-analysis had a much lower replication rate (27% for all associations, 8.6% for associations absent from the largest GWAS) than signals significant in any GWAS (Fig. 3d; **Supplementary Table 3b**). These results suggest that naively meta-analyzing public GWAS within the same ancestry is not a viable strategy – likely because (as might be expected) substantial overlap exists among published studies. We investigated (Methods) whether the limitations of the uncorrected meta-analysis could be mitigated by careful exclusion of clearly overlapping studies but found this to be impractical, as it is often unclear from publications whether a smaller study was a complete or partial subset of a larger study and to what degree: for example, the Global Lipids Genetics Consortium (GLGC) GWAS from 2021^27^ for various lipid traits included 1.5 million individuals from more than 280 cohorts of different ancestries with varying sample sizes.

We therefore investigated a second meta-analysis approach capable of statistically inferring and correcting for sample overlap (“overlap-corrected meta-analysis”) implemented in the METAL software package^14,28^ (Methods). When applied across the 270 trait-ancestry pairs, the overlap-corrected meta-analysis produced 45,272 significant associations, 94% as many as the “any GWAS” approach (Fig. 3e; **Supplementary Table 3a**) and 7.8% more than the largest GWAS. The overlap-corrected meta-analysis not only added associations relative to the largest GWAS (5,867 in total; 21.7 per trait-ancestry pair) but also removed associations (2,609 in total; 9.7 per trait ancestry pair) due to decreases in statistical significance after meta-analysis. Moreover, in the validation analysis, the replication rate of associations unique to the overlap-corrected meta-analysis (55.5%) was significantly higher than the rate for the associations unique to the largest GWAS (40.6%; Fig. 3f; **Supplementary Table 3b**).

We conclude that, among potential approaches to consolidate association evidence across studies into a consensus GWAS, the overlap-corrected meta-analysis is the most viable candidate due to its balance of accuracy and comprehensiveness. In fact, despite being generated by re-analyzing public datasets through a largely automatable pipeline, they have higher expected replication rates than published GWAS produced by bespoke analyst-led analyses.

### Single-ancestry summary statistics from overlap-corrected meta-analysis produce valid downstream results

Beyond enumerating lists of associations, GWAS summary statistics are used in “post-GWAS” bioinformatic methods^6^ that typically make various assumptions about their distribution. Therefore, we explored whether the summary statistics from the overlap-corrected meta-analysis (“overlap-corrected GWAS”) are as suitable for them as are summary statistics from the largest GWAS. First, we focused on association signal fine mapping using COJO^29,30^ (Methods), which seeks to (a) identify independent association signals and (b) calculate probabilities of variant causality. Applied to 191 traits in European ancestry, COJO identified 60,354 “independent signals” (each represented by a significant lead SNP) using the overlap-corrected GWAS and 50,804 independent signals using the largest GWAS. We then merged, ordered, and grouped the independent signals produced by both approaches into 22,317 “shared association regions” (**Supplementary Table 4a;** Methods).

**Figure 4.**
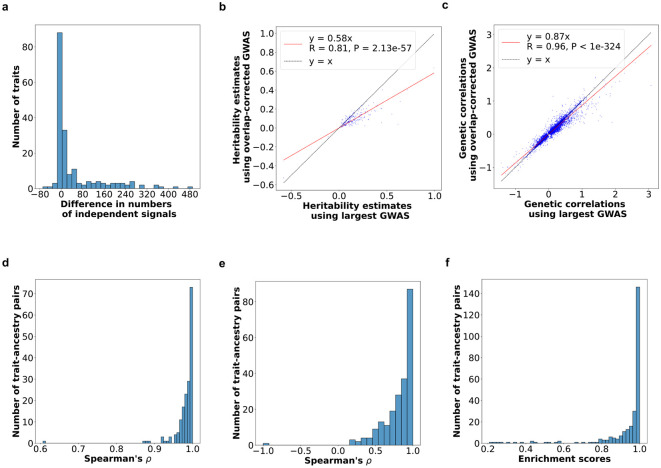
Comparison of single-ancestry polygenic analysis results produced from the overlap-aware GWAS summary statistics versus the largest GWAS summary statistics. **(a)** Number of traits with different numbers of independent signals when using the overlap GWAS versus the largest GWAS. **(b)** Correlation between observed heritability estimates produced by the overlap GWAS and the largest GWAS for 243 trait-ancestry pairs. Black, dashed diagonal lines represent equality; red, solid lines represent linear regressions. **(c)** Correlation between genetic correlation estimates produced by the overlap GWAS and the largest GWAS for 8,097 pairs of traits within the same ancestry. Black, dashed diagonal lines represent equality; red, solid lines represent linear regressions. **(d)** Spearman’s correlation coefficients between tissue-specific functional annotation enrichment produced by the overlap-aware GWAS and the largest GWAS for 174 significant trait-ancestry pairs. **(e)** Spearman’s correlation coefficients between gene-level associations produced by the overlap-aware GWAS and the largest GWAS for 218 significant trait-ancestry pairs. **(f)** Enrichment scores of the top 50 enriched gene sets produced from the largest GWAS in the enriched gene sets produced from the overlap GWAS for 268 trait-ancestry pairs.

In 13,423 (60.1%) of the shared association regions, COJO identified the same number of independent signals when applied separately to the overlap-corrected and largest GWAS. Most of these regions (9,775 regions; 72.8%) yielded only one independent signal in both GWAS. Of the 8,894 (39.9%) regions with different numbers of independent signals, the overlap-corrected GWAS typically produced more signals (Fig. 4a). The increase in independent signals was most pronounced for traits with high degrees of polygenicity and concordant with the increase in GWAS statistical power and decrease in the number of variants in each signal (**Supplementary Fig. 2a; Supplementary Table 4b;** Methods), indicating true independent signals rather than statistical artifacts.

For most of the shared regions between the largest and overlap-corrected GWAS, we identified pairs (one produced by the largest GWAS, and one produced by the overlap-corrected GWAS) of credible sets (each containing one independent signal and all significant variants in LD with it) that completely (76.6%) or at least partially matched (91.6%; Methods). This was true for regions with one credible set produced by each GWAS (95.5% had a complete or partial match) as well as for regions in which multiple credible sets were produced by one GWAS (66.4% had at least a complete or partial match; **Supplementary Fig. 2b**) or both GWAS (98.1% had at least a complete or partial match).

Among the completely matched credible sets, the overlap-corrected GWAS produced credible sets estimated to be 14% smaller than did the largest GWAS (**Supplementary Fig. 2c**). Furthermore, the highest posterior inclusion probabilities (PIPs) in the credible sets produced by the overlap-corrected GWAS were estimated to be 4% greater than their counterparts produced by the largest GWAS (**Supplementary Fig. 2d**). Collectively, these results suggest that fine-mapping association signals by COJO using the summary statistics from the overlap-corrected meta-analysis and largest GWAS are comparable, with differences likely due to the improved fine mapping power of the overlap-corrected GWAS^31,32^.

Second, we evaluated the results from three LD-score regression-based methods to estimate trait heritability^33^, genetic correlations^34^ and tissue-specific functional enrichment^35,36^ when using the overlap-corrected GWAS versus the largest GWAS for 270 trait-ancestry pairs (Methods). We observed similar calibrations of p-values in all three methods (**Supplementary Figs. 3a-c**). Between the two GWAS, we observed highly concordant absolute heritability estimates (Pearson’s correlation coefficient = 0.81, *P* = 2.13×10^−57^; Fig. 4b; **Supplementary Table 5a**), cross-trait genetic correlations (Pearson’s correlation coefficient = 0.96, *P* < 1×10^−324^; Fig. 4c; **Supplementary Table 5b**), and tissue-specific functional enrichments (Spearman’s correlation coefficient > 0.8, *P* < 2.8×10^−4^ for 173 out of 179 trait-ancestry pairs with data available; Fig. 4d; **Supplementary Table 5c**). Absolute heritability estimates were downwardly biased (by nearly 42%) for the overlap-corrected GWAS relative to the largest GWAS (Fig. 4b; **Supplementary Table 5a**), an expected result for genomic control (GC)-corrected GWAS^33^ that we suspect also applies to the standard error inflation procedure used by the overlap-corrected meta-analysis. We observed a much smaller (13%) downward bias in genetic correlations when using the overlap-corrected GWAS summary statistics compared to when using the largest GWAS summary statistics (Fig. 4c; **Supplementary Table 5b**).

Third, we applied MAGMA^37^ to calculate gene-level associations and gene set enrichments from both the overlap-corrected and largest GWAS for 270 trait-ancestry pairs (Methods). Both GWAS produced similarly calibrated gene-level associations and gene set enrichments (**Supplementary Figs. 3d-e**). Gene-level associations were highly concordant globally across most traits (Spearman’s correlation coefficient > 0.8 for 142 out of 218 significant and 244 total trait-ancestry pairs; Fig. 4e; **Supplementary Table 6a**). Gene set enrichments were less concordant globally (Spearman’s correlation coefficient > 0.8 for 67 out of 159 significant and 212 total trait-ancestry pairs; **Supplementary Fig. 3f**; **Supplementary Table 6b**), as expected due to MAGMA’s gene sets’ sensitivity to GWAS sample sizes^38^. However, when focused on the 50 most enriched gene sets produced by the largest GWAS, we found that they were significantly enriched in the results produced by the overlap-corrected GWAS (enrichment score > 0.8 for 245 out of 268 significant and 270 total trait-ancestry pairs; Fig. 4f; **Supplementary Table 6c**; Methods).

Collectively, these results establish that a variety of post-GWAS bioinformatic methods (with the expected exception of absolute heritability estimation from LDSC) have similar calibration and high concordance when applied to the overlap-corrected GWAS compared to the largest GWAS. This further solidifies the potential of using the GWAS produced by the overlap-corrected meta-analysis as the consensus GWAS for a trait within each ancestry.

### Trans-ancestry meta-analyses of overlap-corrected GWAS are comparable to published trans-ancestry GWAS

We next expanded our analyses to evaluate approaches for combining published studies of different ancestries. We identified 131 traits with both (a) published GWAS available for at least two ancestries and (b) for comparison, a published trans-ancestry GWAS (**Supplementary Table 7**). We then ran fixed-effect meta-analysis of the single-ancestry overlap-corrected GWAS and enumerated the significant association signals (Methods). Our meta-analysis produced a total of 7,251 association signals, 75.8% more than the 4,124 association signals in the published trans-ancestry GWAS. It reproduced 3,317 signals (80.4%) in the published GWAS, added 3,934 signals (30 per trait; a 95% increase), and removed 807 signals (6.2 per trait; **Supplementary Figs. 4a-b; Supplementary Table 7**).

To estimate the accuracy of the new signals identified by our meta-analysis, we restricted our comparison to 48 traits for which the published trans-ancestry GWAS had a larger sample size than our meta-analysis, treating the published GWAS as gold standards (**Supplementary Table 7**). Within these 48 traits, 2,628 (78.8%) out of 3,336 association signals produced by our meta-analysis were also present in the published GWAS (**Supplementary Fig. 4; Supplementary Table 7)**, a similar if slightly lower replication rate compared to what we observed in our single-ancestry replication analyses (81.9%).

We obtained similar results when we re-conducted our meta-analysis using a random-effects approach (Methods): as previously observed^39,40^, there was a substantial overlap between random-effects and fixed-effects associations (1,808 signals, accounting for 54.2% of fixed-effects and 65.7% of random-effects signals; **Supplementary Fig. 4; Supplementary Table 7**). The random-effects meta-analysis gained 945 (28.3%) and lost 1,528 (45.8%) association signals relative to the fixed-effect meta-analysis, comparable to the proportion of signals gained (30%) and lost (48.1%) by the fixed-effect meta-analysis relative to the published trans-ancestry GWAS (**Supplementary Fig. 4; Supplementary Table 7**). These results suggest that single-ancestry overlap-corrected GWAS can be combined in a straightforward fixed-effects meta-analysis to produce trans-ancestry association signals comparable with those identified by published trans-ancestry GWAS.

### A resource of consensus GWAS for 1,281 traits produced by the “bottom-line” procedure

**Figure 5.**
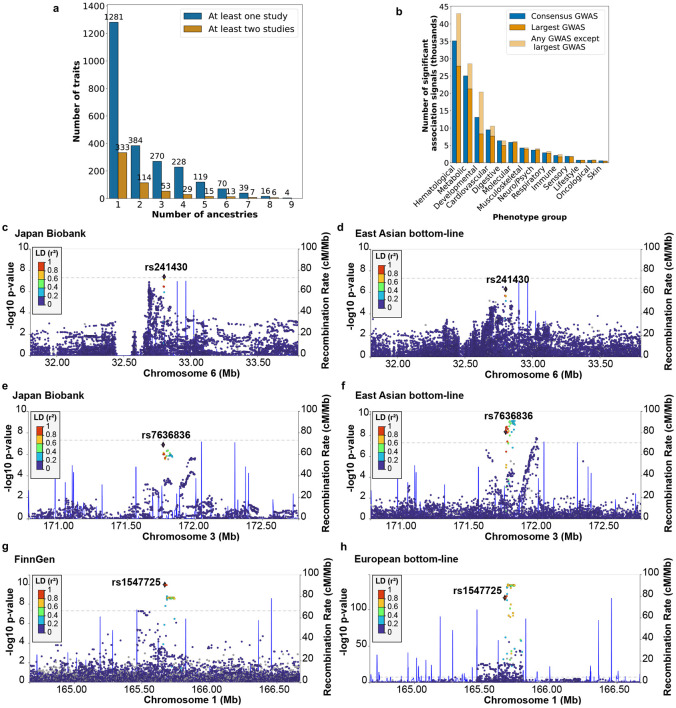
Summary of the resource of consensus GWAS. **(a)** Number of traits with at least one study available in (at least) one to nine ancestries (blue) and number of traits with at least two studies available in (at least) one to nine ancestries (orange) across 1,281 traits in the A2FKP. **(b)** Total number of single-ancestry significant association signals for 870 traits (1,347 trait-ancestry pairs) across 14 phenotype groups produced by the consensus GWAS (blue), the largest GWAS (dark orange), and any GWAS other than the largest GWAS (light orange). Locus zoom plots of the associations in the 2Mb region around rs241430 and POAG produced from the Japan Biobank GWAS **(c)** versus the bottom-line procedure for East Asian ancestry **(d)**. Locus zoom plots of the associations in the 2Mb region around rs7636836 and POAG produced from the Japan Biobank GWAS **(e)** versus the bottom-line procedure for East Asian ancestry **(f)**. Locus zoom plots of the associations in the 2Mb region around rs1547725 and POAG produced from the FinnGen DF1 GWAS **(g)** versus the bottom-line procedure for European ancestry **(h)**.

Based on our results, we developed the “bottom-line” procedure for combining published GWAS summary statistics to generate a single consensus estimate of association for each variant per ancestry and across ancestries for each trait (**Supplementary Fig. 5;** Methods). As of July 2025, we applied the bottom-line procedure to 1,281 traits across 11 disease systems (**Supplementary Fig. 6a**) and 9 ancestries in the A2FKP (**Supplementary Fig. 6b**) using 4,032 datasets from 539 published and 30 unpublished genetic association studies (**Supplementary Tables 8a-c**; Methods). Of these traits, 384 (30%) had studies conducted within different ancestries, 333 (26%) had multiple studies conducted within at least one ancestry, and 205 (16%) had multiple studies within at least one ancestry and studies for multiple ancestries (Fig. 5a). The bottom-line procedure increased sample sizes by 1.73-fold on average compared to the largest GWAS across 426 trait-ancestry pairs (**Supplementary Table 9a)** and produced the first trans-ancestry GWAS for 37 traits (**Supplementary Table 9b**). After LD clumping (Methods), we obtained 111,693 significant association signals spreading across 870 traits (1,347 trait-ancestry pairs) in 14 trait groups for 8 single ancestries (**Supplementary Table 10a**; Fig. 5b). Of the common-variant association signals, 15% were not detected by the largest published GWAS for the trait and 5.8% were never detected by any previous study (**Supplementary Table 10b**). Additionally, we observed 37,715 significant trans-ancestry association signals for 514 traits (**Supplementary Table 10c**).

This resource of consensus GWAS offers a simplified and comprehensive summary of GWAS evidence for each variant and each of these traits. One use is to adjudicate variants with conflicting evidence of association across different studies. Considering one trait, primary open-angle glaucoma (POAG), some variants are significant in one study but in aggregate are not: variant rs241430 was reported as associated with POAG in the Japan Biobank GWAS^41^, but it has since been tested in two other East Asian GWAS which in aggregate fail to replicate the initially reported association (bottom-line *P* = 5.1×10^−7^; Fig. 5c; **Supplementary Table 11**). Other variants become significant only in the bottom-line analysis: variant rs7636836 was not significant in the Japan Biobank GWAS^41^ (*P* = 1.3×10^−7^), but has since has replicated in at least two other GWAS (bottom-line *P* = 4.4×10^−9^; Fig. 5d; **Supplementary Table 11**). Still other variants are significant in all studies but become much more significant in the bottom-line analysis: variant rs1547725 has a European bottom-line *P* = 6.9×10^−117^, which is 55-106 orders of magnitude more significant than reported by any individual GWAS of European ancestry (Fig. 5e; **Supplementary Table 11**).

Another use of the consensus GWAS resource is to solidify genetic support^8^ for genes involved in a trait, including known genes, known genes with no prior genetic evidence, and potentially novel genes. For example, a variant near *LDB3*, rs73344172, was associated with left ventricular ejection fraction (LVEF; *P* = 3.1×10^−9^, beta = 0.063, n = 94,100; **Supplementary Table 11**) in the bottom-line analysis of European ancestry. In previous studies, this variant failed to reach genome-wide significance (**Supplementary Table 11**), although in older genetic studies different variants near *LDB3* were associated with various conditions related to left ventricular dysfunction including left ventricular noncompaction^42^ and hypertrophic cardiomyopathy^43^. The bottom-line associations confirm the association of this variant with LVEF, a finding buttressed by the overlap of rs73344172 with enhancer regions in heart tissue^44,45^, the known function of *LDB3* (it encodes a protein found in both cardiac and skeletal muscle tissues and known for its role in strengthening muscle fibers^46^), and *LDB3*’s high and specific expression in cardiomyocytes^47,48^ (**Supplementary Figs. 7a-c; Supplementary Table 12;** Methods).

In other cases, genes with evidence from non-genetic studies achieve genetic support for the first time in the consensus GWAS. For example, *MSX1*, a gene known for its role in several rare genetic developmental disorders including Wolf-Hirschorn syndrome and Witkop syndrome^49^, was implicated in adipogenesis by a study of *MSX1* knockdown in humans^50^. However, no previous genetic associations were reported between *MSX1* variants and body mass index (BMI) in humans. In the bottom-line analysis, an intronic variant within *MSX1*, rs4689181, was associated with BMI at genome-wide significance (*P* = 6×10^−13^, beta = 0.0092, n = 3,750,730 for European ancestry; **Supplementary Table 11**). This result emphasizes that genes can have genetic support “hidden” across multiple studies that individually do not reach significance (**Supplementary Table 11**). Other examples of this phenomenon include a bottom-line association between rs314274 near *LIN28B* and fasting glucose (**Supplementary Table 11**) that genetically supports previous mouse studies of *Lin28* in glucose metabolism^51-53^, and a bottom-line association between rs28419673 near *ADRA2C* and hypertension (**Supplementary Table 11**) that supports several clinical trials showing *ADRA2C* as a potential therapeutic target for hypertension^54,55^, hypotension^54,56^, and sudden cardiac arrest^54,57^.

Finally, the associations from the consensus GWAS offer numerous new biological leads for various traits. For example, a variant (rs11764290) near *C1GALT1*, a protein coding gene playing a significant role in angiogenesis and thrombopoiesis^58^, reached significance for the first time with CAD (*P* = 1.32×10^−9^, odds ratio = 1.03, n = 1,289,350 for European ancestry; **Supplementary Table 11**). The same and other variants near *C1GALT1* were also associated with blood pressure and high-density lipoprotein cholesterol^27^. Similarly, a variant (rs6916819) near *SESN1*, which encodes for the SESN1 protein which participates in cellular response to DNA damage and oxidative stress^59^, reached significance for rheumatoid arthritis (**Supplementary Table 11**), adding genetic evidence to multiple previous studies showing the crucial involvement of *SESN1* in autophagy^60^ and inflammation^61^, as well as its association with hypothyroidism, an immune condition clinically^62^ and genetically correlated^63^ with rheumatoid arthritis.

**Figure 6.**
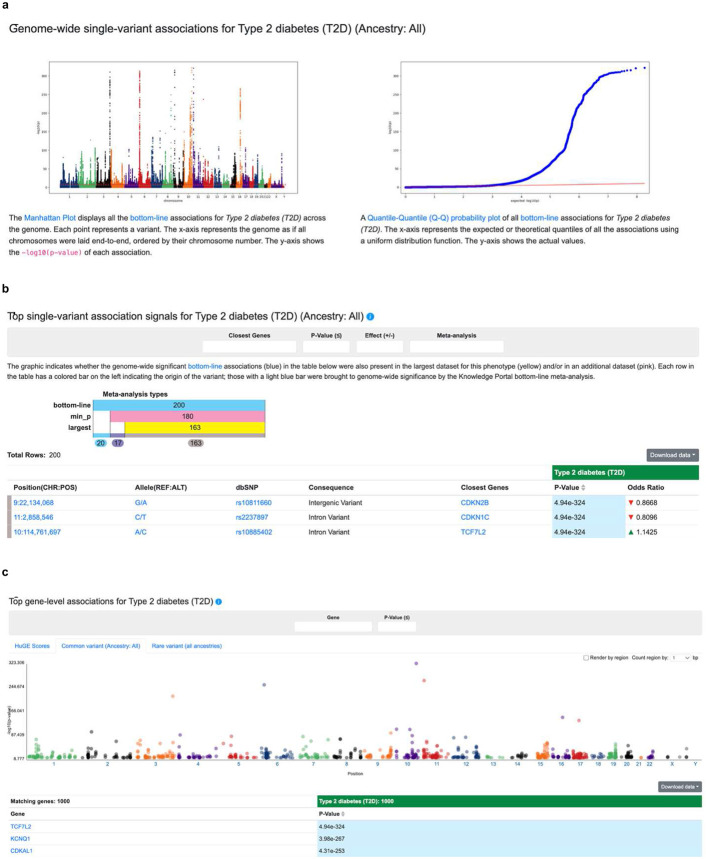
Trans-ancestry bottom-line association and downstream results on the A2FKP for type 2 diabetes (T2D; https://a2f.hugeamp.org/phenotype.html?phenotype=T2D). **(a)** Manhattan plot and QQ plot of the bottom-line GWAS. **(b)** Top variant association signals. **(c)** Gene-level associations produced by MAGMA.

The number of associations within our resource of consensus GWAS that either support previous or suggest new hypotheses is far too large to enumerate in a supplementary table. Therefore, the summary statistics of all consensus GWAS are publicly available on the A2FKP (https://a2f.hugeamp.org) and can be accessed on different pages (for example, the “Phenotype” page; Fig. 6) or via a command line tool (Methods).

## Discussion

In this study, we show that there are many GWAS available for many traits. The existence of multiple GWAS per trait makes seemingly simple tasks such as identifying variants truly associated with the trait or choosing which GWAS to use in downstream analyses challenging. Our results show that existing approaches for estimating the consensus association statistics from published GWAS are either statistically invalid and prone to false positives (using significant estimates from any GWAS for each variant) or discard information (using the largest published GWAS). We apply a novel “bottom-line” approach for combining published GWAS that balances association sensitivity and specificity, produces a single association statistic between each variant and each trait, and yields publicly available consensus GWAS for 1,281 traits. The bottom-line approach is of particular value to researchers who are not experts in adjudicating the strengths and weaknesses of published GWAS – precisely the communities who stand to benefit the most from GWAS reuse. All bottom-line results are publicly available for browsing and download on the A2FKP (https://a2f.hugeamp.org).

The first finding of our study is that widely used techniques for determining whether a variant is associated with a trait are statistically invalid. We suspect that most scientists would find it obvious that excess type I errors will result if a variant’s association statistic is taken as the strongest association ever reported for it, yet researchers implicitly use this approach whenever they search association catalogs or the literature for variant association lists. It is perhaps surprising that our study finds the replication rate of signals produced by “any GWAS” to be only moderately worse than those reported in the largest GWAS, suggesting that (empirically) errors emerging from this approach are not overwhelming. A second finding of our study is that there are a substantial number of undetected associations that are discovered when public studies are combined. In particular, there are numerous traits with trans-ancestry GWAS available for the first time, but we also identify numerous variants which reach single-ancestry significance only after studies are meta-analyzed. In all cases, identifying these new associations requires no new data collection. A third potential impact of our study is that it facilitates the use of GWAS for “reverse genetic” or “genetic support” queries, in which researchers evaluate whether a gene of interest has evidence of association for a trait of interest. The consensus GWAS make the results of these queries unambiguous, avoiding issues in interpretation when a variant is significant in one study but not in others. This is especially useful for experimental scientists who need reliable evidence of GWAS loci to facilitate the conduct of functional studies.

Our study has several limitations. First, when comparing the different consensus estimation approaches, we analyzed only the number of significant association signals produced by each approach, rather than other metrics that may be of interest such as association effect sizes. Second, since it is not possible to know the true association between a variant and a trait, we used the largest GWAS as a proxy for “gold-standard” datasets, which may be conservative. Third, the bottom-line associations produce similarly calibrated and concordant results as compared to the largest published GWAS when applied to several – but not all – commonly used bioinformatic methods. Of the methods that we have not evaluated the bottom-line associations on, some assume independence of samples between datasets (for example, between exposure GWAS and outcome GWAS in Mendelian Randomization, or between training datasets and test datasets in polygenic risk score prediction). In these cases, using the bottom-line associations may not be ideal since they include samples from most published datasets. Fourth, the sample overlap correction procedure of the bottom-line approach does induce some modest downward bias in association estimates, which appears similar to GC-correction commonly used in the GWAS themselves; if this bias is critical for a downstream application, additional research would be necessary to quantify its impacts. Finally, we analyzed only traits in the A2FKP, rather than every published GWAS summary statistic, and our conclusions are most robust with regard to the trait areas covered by the A2FKP. These limitations exist alongside others of GWAS in general, including historical bias towards European populations and focus on common diseases and complex traits (rather than rare diseases).

Nonetheless, bottom-line GWAS on the A2FKP represent a concrete step towards organizing and clarifying the vast amount of information across published GWAS into consensus association estimates for each variant. There has been substantial criticism over the years regarding what GWAS have^64^ (or have not^65^) found, and efforts are now in full force to tease out genes^66^ and biology identifiable from GWAS associations. A comprehensive, harmonized, and single resource of consensus GWAS association statistics is a valuable foundation for such efforts.

## Methods

### Curation of GWAS summary statistics

The A2FKP is a NHGRI funded community genomic resource maintained with the assistance of 16 disease research communities. As of July 2025, the A2FKP consisted of 4,032 summary statistic datasets from 539 published and 30 unpublished genetic association studies (**Supplementary Tables 8a-c)**. Most (86.3%) of these are GWAS summary statistics, and the remainder (13.7%) are summary statistics from other array-based or sequence-based studies. These datasets spanned 1,281 traits, 11 disease systems (**Supplementary Fig. 6a**), and 9 ancestries (**Supplementary Fig. 6b**).

To create a comprehensive database of summary statistics, we curate the datasets in three ways. First, we collaborate with researchers at the European Bioinformatics Institute (EBI) which hosts the GWAS Catalog^5^ to receive published summary statistics at a large scale. We only ingest datasets that have full summary statistics (since many only provide the top associations) and were published after 2018 (since most older studies were either already in our databases or subsumed in larger, more recent studies). Second, to stay up-to-date with the literature, we screen new preprints and publications weekly via PubMed searches and social media platforms such as LinkedIn and X for new association studies. If the summary statistics are publicly available, we download and ingest them into our database; if they are not publicly available, we contact the corresponding authors to request them. Third, we work closely with researchers of various disease communities (for example, researchers studying type 2 diabetes, neurodegenerative disease, or musculoskeletal disease) who contribute their (often unpublished) summary statistics directly. We integrate these datasets into our database and provide public download links as part of the researchers’ manuscripts if requested.

Following these three channels, we update our database with high-quality datasets (full summary statistics with at least chromosome, position, effect allele, non-effect allele, p-value, effect size available for each variant) every four months. We target common diseases, their complications and related traits across 11 physiological systems. Due to the history of the knowledge portals, most of our traits pertain to common metabolic and cardiovascular diseases. Therefore, we have recently sought out phenotypes in other disease areas and datasets of non-European ancestries that are under-represented. Finally, we prioritize datasets from studies that focus on a trait or trait group since they have well-curated phenotype definitions compared to studies that cover many traits (for example, publications of bioinformatic methods that conducted genetic association studies for hundreds of traits).

Since each phenotype and ancestry may be named differently across studies, we carefully read their definitions in each publication or ask the authors if the definitions are not available. We then harmonize the phenotypes to existing ontology labels (EFO and MONDO) if there is a match. For traits without an ontology label match, we create internal identifiers (**Supplementary Table 8b**). For ancestries, we have a fixed set of 9 ancestries commonly represented in the field: African American or Afro-Caribbean, African unspecified, Sub-Saharan African, European, East Asian, South Asian, Hispanic or Latin American, Greater Middle Eastern, and trans-ancestry (**Supplementary Table 8c**). We discard datasets from ancestries (for example, Asian unspecified) that cannot be mapped to any of these labels. Our rigorous process of dataset identification and phenotype selection and harmonization results in a highly selective resource of summary statistics, which likely understates the number of GWAS available for many traits.

For each summary statistics dataset that we obtain, we apply a four-step quality control (QC) procedure. First, we lift over^67^ the variant positions if they are not already in GRCh37 and standardize column names. Second, we flip the alleles (and consequently, the direction of effect) if the non-effect allele is not already the reference allele for each variant. Third, we exclude variants whose statistics are obviously problematic, for example, variants with negative allele frequencies or missing p-values. Fourth, we scale the effect sizes across all studies for each quantitative phenotype so that they are in the same units. More details on this curation and QC process can be found in Costanzo et al. 2023^17^.

Note: all analyses in the Results section except in “A resource of consensus GWAS for 1,281 traits produced by the “bottom-line” procedure” were conducted using datasets in the A2FKP as frozen in July 2024.

### LD clumping to enumerate association signals from GWAS summary statistics

For each set of GWAS summary statistics, we use the LD-clumping method in PLINK^68,69^ to identify the independent association signals. We apply the following parameters: “--clump-p1 5e-8 --clump-p2 5e-6 --clump-r2 0.01 --clump-kb 5000” (which means we only considered variants with GWAS p-value < 5×10^−8^ or 5×10^−6^ as candidates for lead SNPs and merged all variants with r^2^ ≥ 0.01 and within the ± 2.5Mb regions of the lead SNPs into a clump). The r^2^ threshold is set to be lower than the default setting (r^2^ > 0.5) so that the list of association signals produced is overly conservative, and the comparisons between the analyzed consensus estimation approaches (see below) would not be biased due to the abundance of signals in LD with each other. If there is no genome-wide significant association (*P* < 5×10^−8^) for a trait across the entire genome, the value for “clump-p1” (the significance threshold p-value for lead SNPs) is increased in 10-fold increments until there are at least 50 clumps for that trait. The value for “clump-p1” is never set to be higher than the value for “clump-p2”, the secondary significance threshold. For single ancestries, we use LD reference panels from the 1000 Genomes Project^70^. For trans-ancestry GWAS, we first run LD-clumping using the single-ancestry reference panels, and merge clumps across single ancestries which shared at least one variant to obtain trans-ancestry clumps. Rare variants that are not represented in 1000 Genomes and do not fall within the boundaries of existing clumps are appended to the results as single-variant clumps. After LD-clumping, the full summary statistics are reduced to a list of significant “association signals”, each of which is a lead variant that achieved *P* < 5×10^−8^ together with all variants in LD with it. In this manuscript, we used LD clumping to (a) enumerate association signals from summary statistics derived from consensus estimation approaches for single ancestries and trans-ancestry and (b) enumerate association signals produced by the bottom-line procedure in the A2FKP. More details on LD-clumping can be found in Costanzo et al. 2023^17^.

### Estimating the consensus association statistics from published GWAS

#### Single-ancestry analyses

As the existence of multiple GWAS for a trait can lead to challenges in interpreting and re-using GWAS results as well as statistical errors when identifying significant GWAS loci, there is a need to consolidate multiple GWAS into a consensus GWAS. Ideally, datasets are combined via meta-analysis approaches that assume disjoint sets of samples across cohorts – an approach taken by most large-scale GWAS consortia. This requires access to individual-level genotypes and phenotypes (even if these are not shared centrally) and thus statistics can be recomputed after removing overlapping samples. When combining published studies, however, individual-level data are not available and therefore a lack of overlap across studies cannot be assured.

One common use of GWAS summary statistics is to identify significant genomic loci for complex traits. Therefore, we compared different approaches to estimate the consensus association statistics from multiple GWAS in the context of enumerating significant association signals. In each approach, association signals were produced by applying LD-clumping to the full set of p-values of all variants available for each trait-ancestry pair. Since LD reference panels are ancestry-specific and only contain common variants, we restricted this analysis to single-ancestry GWAS for each trait, and variants with MAF > 5%.

##### Main analysis.

In the main analysis, to determine the shared and unique association signals produced by the different combination approaches, we conducted the following process. For each clump in each approach, we constructed a complete graph where each vertex was a unique SNP. Then, we obtained the union of every complete graph coming from each combination approach where any two complete graphs were connected via shared SNP vertices. Next, we ran connected component analysis where each connected component was a unique signal and labelled each signal according to which combination approaches produced it. This, in effect, considers two association signals from two approaches to be the same if their intersection was non-empty. Therefore, the number of “merged” significant association signals could be less than the original number of significant association signals within and between approaches. Finally, we counted the number of association signals shared by and unique to the consensus estimation approaches. From 830 traits and 9 ancestries (2,602 summary statistics datasets in total) available in the A2FKP as of July 2024, we restricted this analysis to 270 trait-ancestry pairs (across 207 traits and 7 ancestries) that had summary statistics from at least two studies (**Supplementary Table 3a**).

##### Validation analysis.

To evaluate whether the signals produced by each of the four approaches (see below) were true or false positives, we conducted a retrospective leave-one-out validation analysis as follows. For each trait-ancestry pair, we removed the largest GWAS and used it as a “gold-standard” dataset with “gold-standard” significant association signals (**Supplementary Table 3b**). As a result, the second largest GWAS became the largest GWAS. The analysis was, therefore, restricted to 97 trait-ancestry pairs (across 80 traits and 5 ancestries) with at least two remaining studies. In each consensus estimation approach, we conducted the listing of association signals but with the remaining datasets (without the gold-standard dataset). We considered an association signal a true positive if it was also significant in the gold-standard GWAS, and a false positive if it was not significant in the gold-standard GWAS. We defined the replication rate (or accuracy) for each list of association signals as the ratio of the number of true positives over the total number of signals.

##### Approach 1: “Any GWAS”.

One common approach is to search the literature or catalogs of GWAS associations^5^ for any significant association reported nearby the gene. This is equivalent to considering a variant to be significantly associated with a trait if any GWAS at all produced a significant estimate for the variant. This approach overlooks all studies that show non-significance and produces summary statistics that comes from a variety of GWAS rather than a cohesive one; therefore, it is statistically invalid. In our analyses, to recreate this approach, we assigned each variant-trait association the estimate with the lowest p-value across all studies, regardless of which study produced this estimate. We showed that enumerating association signals using significant estimates from any and all GWAS, as hypothesized, introduced excess false positives (Fig. 3b; **Supplementary Fig. 1c**).

##### Approach 2: Largest GWAS.

Another widely used option is to default to the GWAS with the largest sample size under the assumption that it is the most comprehensive. In our analyses, we assigned each variant-trait association the estimate from the GWAS with the largest sample size. This is equivalent to using the full summary statistics from the largest GWAS for each trait. We showed that although the largest GWAS were the most comprehensive in terms of samples but not necessarily the most comprehensive in terms of significant signals for two reasons. First, many significant signals detected by other GWAS were not present in the largest GWAS which can happen due to variant filtering^7^; **Supplementary Fig. 1b**). Second, the largest GWAS simply did not produce a significant estimate for a variant that was significant in other GWAS which indeed turned out to be a true signal rather than statistical noise (**Supplementary Fig. 1b**). Therefore, even though enumerating association signals considering only the largest GWAS reduced false positives relative to accepting any significant estimate from any GWAS, the largest GWAS is conservative and more prone to false negatives.

##### Approach 3: Uncorrected meta-analysis.

To address the statistical limitations of the two approaches above, we asked whether we could simply meta-analyze all published studies. Such a meta-analysis could be effective if published studies have no or minimal sample overlap. We termed this approach the “uncorrected meta-analysis” due to the ignorance of sample overlap. We implemented the uncorrected meta-analysis using the summary statistics from all studies in METAL^28,71^ with the parameter “OVERLAP OFF”. The uncorrected GWAS produced a much larger number of association signals relative to the largest GWAS and any GWAS (Fig. 3c; **Supplementary Table 8a**). However, most of these signals were false positives (Fig. 3d; **Supplementary Table 8b**). These results suggest that perhaps as expected, many GWAS have a high degree of sample overlap, and naively meta-analyzing them without overlap correction leads to significant inflation of statistics and consequently, an unacceptably high rate of false positives.

We therefore attempted to manually identify and remove datasets whose samples were subsumed in other datasets before meta-analyzing. We first looked for obvious indications in the dataset IDs. For example, “GWAS_UKBiobankFatDist_eu_females” and “GWAS_UKBiobankFatDist_eu_males” were complete subsets of “GWAS_UKBiobankFatDist_eu” due to having only either male or female samples from the main dataset (PMID: 30664634^72^), and therefore removed. Then, for more complicated datasets that were meta-analyses of other studies such as “Mahajan2022_T2D_EU” (T2D GWAS from PMID: 35551307^72^), we thoroughly went through the portal website (in this case, https://md.hugeamp.org/) and checked the list of datasets were included into the meta-analysis and if those datasets existed in our list, we excluded them from the meta-analysis. For datasets with no such list, we checked the original paper and supplementary tables to find out the list of studies/cohorts used and excluded their summary statistics datasets from our meta-analysis. However, in most cases, it was challenging to identify specific redundancies in samples, as many GWAS were conducted on different cohorts or combinations of them, with unknown degree of overlap^11,12^. For example, the Global Lipids Genetics Consortium (GLGC) GWAS from 2021^27^ included ~1.3M samples of European ancestry from 159 cohorts, >225K of which were from the Million Veteran Program (MVP)^73^ whereas the most recent GWAS for the same traits by MVP^74^ contained >390K samples of European ancestry. If we had removed the MVP GWAS, we would miss out on the MVP samples not included in the GLGC GWAS, and vice versa, if we had removed the GLGC GWAS, we would miss out on the non-MVP samples. Furthermore, since this is a manual process requiring careful cataloging of all datasets and the samples that were included in them, it is not practical on a large scale (for thousands of traits and datasets).

##### Approach 4: Overlap-corrected meta-analysis.

We investigated a second meta-analysis approach termed “overlap-corrected meta-analysis”. In this approach, we conducted meta-analysis of the summary statistics from all studies using METAL with the parameter “OVERLAP ON” to statistically infer and correct for sample overlap among the studies. Briefly, this approach uses empirically determined covariances of association statistics between studies to adjust association statistic standard errors and consequently, p-values to prevent over-inflation that would have occurred with “OVERLAP OFF” in the uncorrected meta-analysis. We showed that the overlap-corrected meta-analysis produced lists of association signals that were more comprehensive than signals produced by the largest GWAS (Fig. 3e; **Supplementary Table 8a**), and more accurate than signals produced by either the largest GWAS or using significant statistics from any GWAS (Fig. 3f; **Supplementary Table 8b**).

#### Trans-ancestry analyses

As many recent GWAS emphasize contributions of samples of multiple ancestries, we next evaluated approaches for combining published studies of different ancestries. Similarly to the single-ancestry analyses, we compared the lists of association signals produced by two approaches. The first approach was to use published trans-ancestry GWAS with the largest sample size. The second approach was a fixed-effects meta-analysis of single-ancestry overlap-corrected GWAS for traits with at least two ancestries available. Since the sample overlap was already accounted for within each single-ancestry meta-analyzed GWAS, and there were presumably no overlapping samples across ancestries, we applied METAL^28,71^ assuming no overlap, that is, with the parameter “OVERLAP OFF”.

##### Main analysis.

In the largest published trans-ancestry GWAS and our trans-ancestry meta-analysis, we merged clumps across all ancestries into one clump if they had any overlapping SNPs. We then enumerated significant association signals from each trans-ancestry GWAS and determined whether two signals from two GWAS (for a trait) were the same signal following the method used in the single-ancestry analyses. From 830 traits available in the A2FKP as of July 2024, we restricted this analysis to 131 traits with both (a) published GWAS available for at least two ancestries and (b) a published trans-ancestry GWAS (**Supplementary Table 7**). We found that our meta-analysis produced 75.8% more signals than the published GWAS and reproduced 80.4% signals identified in the published GWAS (**Supplementary Fig. 4a; Supplementary Table 7**). As a sensitivity analysis, we repeated the same analysis using the European reference panel for clumping, since the majority of samples were likely of European ancestry and observed similar patterns of overlap between the published GWAS and our trans-ancestry GWAS (**Supplementary Fig. 4b**).

##### Validation analysis.

Similarly to the single-ancestry analysis, we evaluated the accuracy of the lists of association signals produced by our trans-ancestry meta-analysis. We considered the gold-standard dataset for each trait as the largest published trans-ancestry GWAS if its sample size was larger than or equal to our trans-ancestry meta-analysis, restricting the validation analysis to 48 traits (**Supplementary Table 7**). We found that 78.8% of signals in the fixed-effects meta-analysis were replicated in the published GWAS (**Supplementary Fig. 4c; Supplementary Table 7**). Since many trans-ancestry GWAS employed a random-effects model as a follow-up to the fixed-effects model to account for allelic heterogeneity^75^, we repeated this analysis with a random-effects meta-analysis for the 48 traits. To obtain the random-effects GWAS, we applied MR-MEGA^75^ to the single-ancestry overlap-corrected GWAS with the default parameters. Consistent with previous observations^39,40^, we found substantial overlap of significant association signals between the fixed-effects and random-effects meta-analyses (**Supplementary Fig. 4; Supplementary Table 7**).

### Polygenic analyses of GWAS summary statistics

There are many downstream uses of GWAS summary statistics, including to (a) fine-map association signals^30^, (b) estimate complex trait heritability^33^, (c) estimate cross-trait genetic correlations^34^, (d) calculate enrichment of tissues^35,36^, genes and gene sets^37^ for complex trait associations. These post-GWAS analysis methods typically make stronger assumptions about the summary statistics distribution than do methods that simply clump variants into association signals. We therefore explored whether the summary statistics produced by the single-ancestry overlap-corrected meta-analysis were suitable for use in such methods in comparison to the summary statistics from the largest GWAS.

#### Conditional and fine-mapping analyses using COJO**

We first focused on association signal fine mapping, which seeks to (a) identify independent association signals through conditional analysis and (b) calculate posterior probabilities of variant causality. In practice, fine-mapping approaches are a more sophisticated alternative to the clumping approach we applied for our association signal analysis, and there are concerns that significant errors can be introduced by mismatches between LD as measured in the study samples versus the reference panel used for analysis – concerns that could be plausibly higher given the approximations made by the overlap-corrected approach. To investigate these concerns, we applied the fine mapping method COJO^30^, commonly used in automated fine mapping databases^76^, to 191 traits in European ancestry (only the European ancestry GWAS had sufficient data for this analysis; **Supplementary Table 4a**). We chose this method for comparison purposes due to its simplicity; many fine-mapping approaches include additional steps such as the use of variant annotations to characterize potential novel causal variants.

##### COJO implementation.

For the conditional and fine-mapping analyses, we applied the following parameters: “--maf 0.01 --cojo-p 5e-08 --cojo-wind 2000 --cojo-collinear 0.9 --cojo-slct” (which means we only considered variants with MAF >1% and GWAS p-value < 5×10^−8^ as candidates for lead SNPs, examined the variants in ± 1Mb region around the lead SNPs, removed highly correlated lead SNPs to avoid collinearity, and used the stepwise model selection procedure). These analyses resulted in 60,354 “independent signals” (each represented by a significant lead SNP) using the overlap-corrected GWAS and 50,804 independent signals using the largest GWAS across 191 traits.

##### Enumeration of shared association regions.

Since different GWAS inherently produce different GWAS association signals, we grouped the identified signals into genomic regions and compare the signals within the same region. Therefore, we retrospectively assigned the independent signals to contiguous chromosomal regions as follows: (1) for each trait, get a list of all lead SNPs from both GWAS, (2) merge the two lists of lead SNPs into one list and sort the lead SNPs by chromosome and position, (3) enumerate the first lead SNP’s position as the start of the first association region, (4) for each next SNP in the list, if it is less than 1Mb from the start of the current region, assign to the current region and if not, enumerate the next region with the position of the SNP as the start until all lead SNPs have been assigned to a region. This procedure produced 28,374 regions (across 191 traits), 22,317 of which were considered “shared association regions” (across 188 traits) as they contained at least one independent signal from each GWAS (**Supplementary Table 4a)**.

In 13,423 (60.1%) of the shared association regions, COJO identified the same number of independent signals when applied separately to the overlap-aware and largest associations. Most of these regions (9,775 regions; 72.8%) yielded only one independent signal in both the overlap-aware and largest analyses. Of the 8,894 (39.9%) regions with different numbers of independent signals, the overlap-aware approach typically produced more signals (average number of additional signals = 1.6, SD = 1.2 across 7,538 regions). These results held when we accumulated the regions on the trait level (**Supplementary Table 4b**). However, we observed a difference in the number of independent signals in drastically more shared regions for some traits than others — the top 40 traits for which the two approaches identified different numbers of independent signals for more than 100 shared regions (the overlap-aware approach identified more for 39 of these traits) belong to hematological, anthropometric and cardio-metabolic phenotype groups exclusively, likely due to their high levels of polygenicity and GWAS statistical power (**Supplementary Table 4b**). Collectively, the overlap-aware approach identified more signals for most traits than did the largest (Fig. 4a), which is moderately concordant with the increase in sample sizes (Pearson’s correlation coefficient = 0.36, *P* = 4.4×10^−7^; **Supplementary Fig. 2a**; **Supplementary Table 4b**). Among the 10 traits with the largest fold increases in the number of independent signals by the overlap-aware approach, 8 had sample sizes at least 1.7-fold higher (**Supplementary Table 4b**). Furthermore, we observed a decrease in the size of the independent signals with the median dropping from 7 variants in the largest approach to 4 variants in the overlap-aware approach. These results are consistent with previous predictions^31^, supporting that these additional signals are likely real.

##### Evaluation of credible set matching.

For each shared region for each trait, we evaluated to what extent the two GWAS shared causal variant predictions via the calculations of “credible sets”, each of which contained a lead SNP and other SNPs in LD with the lead SNP. In each credible set, the sum of the posterior inclusion probabilities (PIP) for all variants was at least 0.99. For each credible set produced by the largest GWAS, we matched it with each of credible sets (in the same region) produced by the overlap-corrected GWAS, and extracted the SNPs shared between each pair. Then, we calculated the sum of PIPs of the shared SNPs for each GWAS and defined the smaller sum as the “shared PIP sum”. We defined the two credible sets as “complete match” if their shared PIP sum > 0.6, “partial match” if 0 < their shared PIP sum ≤ 0.6, and “non-match” if their shared PIP sum = 0.

In most cases, we identified pairs of credible sets (one produced by the largest GWAS, and one produced by the overlap-corrected GWAS) that completely (76.6% of regions) or at least partially (91.6% of regions) matched. Beginning first with the simplest 9,775 shared association regions with one credible set identified by both the overlap-corrected GWAS and the largest GWAS, 8,048 (82.3%) regions had a complete match of credible sets, and 1,281 (13.2%) regions had a partial match of credible sets. Second, among the 3,799 shared regions in which one of the two GWAS identified one credible set, but the other identified more than one credible set, we identified a complete match of the lone credible set in 1,416 (37.3%) regions. Of these regions, the GWAS with more than one credible set usually produced one credible set that was a complete match and additional credible sets that appeared to be independent (1,223 regions), although there were some cases where the lone credible set appeared to “split” into separate credible sets (193 regions). Of the 2,383 regions without a complete match, 1,106 (46.4%) had at least one partial match (**Supplementary Fig. 2b**). Third and finally, the most complex shared association regions (multiple credible sets produced by each GWAS) typically did have matching credible sets: 7,633 (87.3%) of these 8,743 regions contained at least one complete match, 8,581 (98.1%) contained at least one complete or partial match, and 2,866 (32.8%) contained a complete match for each credible set produced by the GWAS with a smaller number of credible sets.

##### Comparison of matched credible sets.

To complete our fine-mapping analysis, we directly compared the credible sets produced by the overlap-corrected and largest GWAS that were complete matches. We found 26,564 pairs of credible sets that were complete matches between the two GWAS across 186 traits, accounting for 57.3% of credible sets identified by the largest GWAS and 47.5% by the overlap-corrected GWAS. Among these pairs, the overlap-corrected GWAS on average produced credible sets that were 14% smaller than did the largest GWAS (**Supplementary Fig. 2**), with most (90%) of their variants typically identified by the largest GWAS. Furthermore, the highest PIPs in the credible sets produced by the overlap-corrected GWAS were on average 4% greater than their counterparts produced by the largest GWAS (**Supplementary Fig. 2d**). Collectively, these results suggest that fine-mapping association signals by COJO using the summary statistics from the overlap-corrected GWAS vs. the largest GWAS are comparable, with the former possibly producing credible sets of higher resolutions^32^.

#### Polygenic architecture analyses using LD score regression (LDSC) methods

To evaluate whether the summary statistics from the overlap-corrected meta-analysis could be used to estimate complex traits’ polygenic architecture, we conducted single trait SNP-based heritability estimation using basic LD-score regression (LDSC)^33^, genetic correlations using cross-trait LDSC^34^, and tissue-specific functional enrichment using stratified LDSC^35,36^ with the overlap-corrected GWAS and the largest GWAS for 270 trait-ancestry pairs. For all three methods, we used LD scores from 1000 Genomes Project^70^. More implementation details can be found at https://zenodo.org/records/7545715.

##### Absolute heritability estimation.

We observed similar distributions of p-values produced by LDSC using either the largest GWAS or the overlap-corrected GWAS (**Supplementary Fig. 3a**). Of the 270 trait-ancestry pairs, 243 had a nominally significant (*P* < 0.05) heritability estimate $\left(h^{2}\right)$ on the observed scale, produced by either the largest GWAS or the overlap-corrected GWAS (**Supplementary Table 5a**). We only considered the significant estimates for further analyses since non-significant estimates could reflect low-powered GWAS and/or poor polygenic architecture^77^. To assess how concordant the estimates were between the two GWAS, we applied Pearson correlation to the paired $h^{2}$ values of 243 trait-ancestry pairs (**Supplementary Table 5a**). We found a significant and positive correlation between the estimates from the overlap-corrected and the largest GWAS (Pearson’s r = 0.81, *P* = 2.13×10^−57^; Fig. 4b). To assess the bias in $h^{2}$ values produced by the overlap-corrected GWAS relative to the largest GWAS, we linearly regressed the $h^{2}$ values produced by the overlap-corrected GWAS on the $h^{2}$ values produced by the largest GWAS with a fixed intercept = 0. We found a downward bias of 42% in the $h^{2}$ values produced by the overlap-corrected GWAS (Fig. 4b).

##### Cross-trait genetic correlation estimation.

Of the 270 trait-ancestry pairs, 18 pairs had calculated heritability values outside of the recommended bounds for analysis producing invalid genetic correlation results using either the largest GWAS or overlap-corrected GWAS. Of the remaining 252 pairs, we observed similar distributions of p-values across 20,199 unique same-ancestry trait pairs produced by cross-trait LDSC using either the largest GWAS or the overlap-corrected GWAS (**Supplementary Fig. 3b**). Of these pairs, 8,097 had a nominally significant (*P* < 0.05) genetic correlation estimate $\left(r_{g}\right)$ produced by either the largest GWAS or the overlap-corrected GWAS (**Supplementary Table 5b)**. Similarly to LDSC, we only considered the significant estimates for further analyses. To assess how concordant the estimates were between the two GWAS, we applied Pearson correlation to the paired $r_{g}$ values of 8,097 pairs of same-ancestry traits (**Supplementary Table 5b**). We found a significant and positive correlation between the estimates from the overlap-corrected and the largest GWAS (Pearson’s r = 0.96, *P* < 1×10^−324^; Fig. 4c). To assess the bias in $r_{g}$ values produced by the overlap-corrected GWAS relative to the largest GWAS, we linearly regressed the $r_{g}$ values produced by the overlap-corrected GWAS on the $r_{g}$ values produced by the largest GWAS with a fixed intercept = 0. We found a downward bias of 13% in the $h^{2}$ values produced by the overlap-corrected GWAS (Fig. 4c).

##### Tissue-specific functional enrichment.

We obtained ~5K functional genomic annotation datasets from the Common Metabolic Diseases Genome Atlas (CMDGA; https://cmdga.org). We then merged these datasets into 171 annotations across 48 tissues and four different annotation types (accessible chromatin, enhancers, promoters, and binding sites). In total, for each of the 171 annotations, each trait-ancestry pair had two functional enrichment estimates, one calculated from the largest GWAS and one calculated from the overlap-corrected GWAS.

We observed similar distributions of p-values across 46,170 annotation-trait-ancestry combinations produced by stratified LDSC using either the largest GWAS or the overlap-corrected (**Supplementary Fig. 3**). We applied FDR correction to the p-values of the annotations within each trait-ancestry pair. Since the developers of stratified LDSC recommended prioritizing annotations for which FDR < 0.05^35,36^, for each trait-ancestry pair, we only considered annotations with FDR < 0.05 produced by either the largest GWAS or the overlap-corrected GWAS for further analyses. We also removed annotations with absolute values of enrichment beta > 100 as a quality control measure. 179 trait-ancestry pairs remained with at least two significant annotations (**Supplementary Table 5c**). Since stratified LDSC is used to prioritize annotations^35,36^, rather than estimate the exact magnitude of heritability, for each trait-ancestry pair, we applied Spearman’s rank correlation to the enrichment betas of the significant annotations to assess global concordance. We found that 174 trait-ancestry pairs had a significant and positive correlation (*P* < 2.8×10^−4^ due to Bonferroni correction for 179 tests; Fig. 4d; **Supplementary Table 5c**). Of these, 173 pairs had a correlation coefficient ρ>0.8, indicating substantial global concordance between the annotations prioritized for each trait in each ancestry using the summary statistics from either the largest GWAS or the overlap-corrected GWAS. The two traits in European ancestry that had ρ<0.8 were chronic kidney disease (CKD; ρ=0.42, *P* = 8.6×10^−4^) and waist circumference (WC; ρ=0.61, *P* = 5.7×10^−11^).

#### Gene and gene set prioritization using MAGMA

To evaluate how well the summary statistics from the overlap-corrected meta-analysis could identify relevant genes and genes sets relative to the summary statistics from the largest GWAS, we conducted gene-level association and gene set enrichment analyses using MAGMA^37^ using the two GWAS separately for 270 trait-ancestry pairs. More implementation details can be found at https://zenodo.org/records/7545715.

##### Gene-level associations.

We included 19,241 genes in our analyses. We observed similar distributions of p-values across 4,843,293 gene-trait-ancestry combinations produced by MAGMA using either the largest GWAS or the overlap-corrected (**Supplementary Fig. 3d**). We applied FDR correction to the p-values of genes within each trait-ancestry pair. Since MAGMA only outputs p-values which are used to rank the genes, we applied Spearman’s rank correlation to the significant genes (FDR < 0.05), ranking by original p-values, produced for each trait-ancestry pair using the summary statistics from either GWAS. Of the 244 trait-ancestry pairs with at least two significant genes, 218 had a significant correlation (*P* < 2×10^−4^ due to Bonferroni correction for 244 tests; Fig. 4e; **Supplementary Table 6a**). Of these, 142 pairs had a correlation coefficient ρ>0.8, indicating substantial global concordance between the annotations prioritized for each trait in each ancestry using the summary statistics from either the largest GWAS or the overlap-corrected GWAS. One trait in European ancestry that had a significant negative correlation was leptin due to the reverse rankings of two genes in the two GWAS (ρ=−1, *P* < 1×10^−324^, number of genes = 2).

##### Gene set enrichment.

We used 21,815 gene sets from the Molecular Signatures Database (MSigDB)^78^. We observed similar distributions of p-values across 5,876,581 gene set-trait-ancestry combinations produced by MAGMA using either the largest GWAS or the overlap-corrected (**Supplementary Fig. 3e**). We conducted global concordance analysis to the gene set enrichment following the same process for gene-level associations. We found that 212 trait-ancestry pairs had at least two significant gene sets (FDR < 0.05 using either of the two GWAS). Of these, 159 pairs had a significant correlation (*P* < 2.4×10^−4^ due to Bonferroni correction for 212 tests), and 67 had a significant correlation with ρ>0.8 (**Supplementary Fig. 3f; Supplementary Table 6b**). The lower global concordance in MAGMA gene set results relative to the MAGMA gene results was expected since MAGMA gene set results are sensitive to GWAS sample sizes^38^.

Since researchers are less likely to consider gene sets beyond the top ones, we conducted an additional analysis to evaluate the enrichment of the top 50 gene sets prioritized using the largest GWAS in the full gene set enrichment results produced from the overlap-corrected GWAS. We implemented a similar method to GSEA^79^ as follows: (1) for each trait-ancestry pair, rank all the gene sets by the p-values produced by MAGMA using the largest summary statistics from lowest (most prioritized) to highest (least prioritized), (2) define the observed reference set of gene sets as the top 50 gene sets prioritized by MAGMA using the overlap-corrected summary statistics, (3) compute the observed enrichment walk score, (4) create 1000 alternative reference sets by randomly sampling 50 gene sets 1000 times and compute 1000 permuted enrichment scores (i.e., the null distribution of enrichment scores), (5) calculate the p-value of the observed score based on the null distribution and apply FDR correction across all trait-ancestry pairs. An enrichment score is considered significant if FDR < 0.05. We found that in most cases, the top gene sets prioritized using the largest GWAS were significantly enriched in the gene set results produced from the overlap-corrected GWAS: 268 trait-ancestry pairs had a significant enrichment score, 245 of which had an enrichment score > 0.8 (Fig. 4f; **Supplementary Table 6c**).

##### “Bottom-line” procedure

We apply the bottom-line procedure to each trait. First, we separate the genetic association datasets into either single-ancestry (African American or Afro-Caribbean, African unspecified, East Asian, European, Greater Middle Eastern, Hispanic or Latin American, South Asian, and Sub-Saharan African) or trans-ancestry (multiple ancestries in the same GWAS). For each dataset, we remove multiallelic variants and variants with missing p-values, or effect sizes (or odds ratio). We partition the remaining variants in the single-ancestry datasets into common (MAF > 5%) and low-frequency/rare (MAF ≤ 5%) variants. For common variants, we run METAL^28,71^ in all datasets of each ancestry with the parameter “OVERLAP ON” (i.e., overlap-corrected meta-analysis). For low-frequency/rare variants, we only keep the association statistics from the largest dataset. We then concatenate the outputs for common and low-frequency/rare variants. In the event that a variant exists in both MAF categories (from different datasets), we only keep the statistics from the largest dataset (**Supplementary Fig. 5**).

After we process each ancestry, we apply METAL across all the ancestries with the parameter “OVERLAP OFF”. For each variant, we keep the association statistics from either the trans-ancestry meta-analysis or the largest published trans-ancestry study, whichever has a larger sample size. If no single-ancestry datasets exist for a phenotype, we simply display the results from the trans-ancestry datasets. We term this approach as the “bottom-line” procedure, as for each phenotype, it provides a single estimate of association for each variant in each ancestry, as well as a single estimate of association for each variant across all ancestries (**Supplementary Fig. 5**).

To support the association between *LDB3* and left ventricular ejection fraction, we re-analyzed SCP498 (adult human heart; PMID: 32403949^48^) using flat files from the Single Cell Portal^47^ (https://singlecell.broadinstitute.org/single_cell/study/SCP498/): gene_sorted-matrix.mtx (gene expression matrix), genes_v2.tsv (features), barcodes.tsv (cells), meta.data.v3.txt (metadata), and umap.v2.txt (UMAP and cluster labels). Gene expression values were treated on the portal scale, log(expr/total × 10,000); cells with *LDB3* > 0 were considered expressing. UMAP coordinates and cluster annotations were taken as provided by the original study. Analyses were performed in R using Seurat (v5.3.0)^80^, SingleR (v1.8.0)^81^, and celldex (v1.4.0)^81^. To assess cell-type enrichment, we permuted the *LDB3* expression vector across all cells 1,000 times (cluster membership fixed) and, for each cluster, calculated a permutation p-value as the fraction of permutations with an equal or greater proportion of expressing cells than observed. We annotated 287,269 cells into 17 clusters (**Supplementary Figs. 7a-b**). *LDB3* expression showed myocardial specificity, with high signal in atrial and ventricular cardiomyocytes and minimal expression in stromal, endothelial, and immune lineages (**Supplementary Figs. 7a-b**). A permutation test with 1,000 shuffles confirmed significant enrichment of *LDB3* in cardiomyocytes (*P* < 0.001), whereas non-myocyte clusters showed no enrichment (**Supplementary Fig. 7c; Supplementary Table 12**).

### How to access the bottom-line associations on the A2FKP

First, all summary statistics can be downloaded directly from the AWS Open Data bucket “s3://dig-open-bottom-line-results”. For each phenotype “p” and ancestry “a”, the full bottom-line summary statistics can be downloaded using the following command: “aws s3 –no-sign-request cp s3://dig-open-bottom-line-analysis/bottom-line/$a/$p.sumstats.tsv.gz $download_location”. The full lists of all phenotype IDs and ancestry IDs available on the A2FKP as of July 2025 can be found in **Supplementary Tables 8b-c**.

Second, researchers can browse bottom-line associations by searching for a variant, region, or phenotype of interest on the “Home” page of the A2FKP. Variant-first searches (for example, rs1260326) will lead to the “Variant” page (https://a2f.hugeamp.org/variant.html?variant=2%3A27730940%3AT%3AC) where phenome-wide associations (PheWAS) with a given variant are displayed in a PheWAS plot and table. Region-first searches (for example, chr9:21940000-22190000) will lead to the “Region” page (https://a2f.hugeamp.org/region.html?chr=9&end=22190000&phenotype=CAD&start=21940000) where the genome-wide significant clumped associations (association signals) within a given region are shown in a PheWAS plot and table. A LocusZoom plot is also available if researchers enter a phenotype in the “Genomic Region Miner” of the “Region” page. Phenotype-first searches (for example, heart failure) will lead to the “Phenotype” page (https://a2f.hugeamp.org/phenotype.html?phenotype=HF) where a Manhattan plot and a quantile-quantile plot are displayed to summarize the bottom-line associations with a given phenotype. Below these two plots is a table showing the top 1000 significant association signals for the phenotype. On the “Phenotype” page, researchers can also see the results from bioinformatic methods applied to the bottom-line associations, including tissue-specific functional enrichment, cross-trait genetic correlations, gene-level associations, and gene set enrichment. The default ancestry for all results is trans-ancestry (displayed as “All ancestries”); however, researchers can choose a different ancestry from the drop-down menu “Set Ancestry” at the top of any page. More documentation is available on the “Help” page of the A2FKP (https://a2f.hugeamp.org/help.html).

## Supplementary Material

This is a list of supplementary files associated with this preprint. Click to download.

- BLpaperSuppTablesJan2026.xlsx
- BLpaperSuppFiguresJan2026.pdf

## Data Availability

All summary statistics are publicly available for browsing on the Association to Function Knowledge Portal (https://a2f.hugeamp.org) and download from the AWS Open Data bucket “s3://dig-open-bottom-line-results”.

## Code Availability

All original code is available at https://zenodo.org/records/7551651.
